# Jacob, a Synapto-Nuclear Protein Messenger Linking N-methyl-D-aspartate Receptor Activation to Nuclear Gene Expression

**DOI:** 10.3389/fnsyn.2021.787494

**Published:** 2021-11-26

**Authors:** Katarzyna M. Grochowska, Julia Bär, Guilherme M. Gomes, Michael R. Kreutz, Anna Karpova

**Affiliations:** ^1^Research Group (RG) Neuroplasticity, Leibniz Institute for Neurobiology, Magdeburg, Germany; ^2^Leibniz Group ‘Dendritic Organelles and Synaptic Function’, University Medical Center Hamburg-Eppendorf, Center for Molecular Neurobiology Hamburg, Hamburg, Germany; ^3^Research Group (RG) Neuronal Protein Transport, University Medical Center Hamburg-Eppendorf, Center for Molecular Neurobiology Hamburg, Hamburg, Germany; ^4^Research Group (RG) Optobiology, Institute of Biology, HU Berlin, Berlin, Germany; ^5^Center for Behavioral Brain Sciences (CBBS), Magdeburg, Germany; ^6^German Research Center for Neurodegenerative Diseases (DZNE), Magdeburg, Germany

**Keywords:** Jacob/*NSMF*, CREB, NMDAR, nuclear localization signal (NLS), importin-α1, synaptic plasticity

## Abstract

Pyramidal neurons exhibit a complex dendritic tree that is decorated by a huge number of spine synapses receiving excitatory input. Synaptic signals not only act locally but are also conveyed to the nucleus of the postsynaptic neuron to regulate gene expression. This raises the question of how the spatio-temporal integration of synaptic inputs is accomplished at the genomic level and which molecular mechanisms are involved. Protein transport from synapse to nucleus has been shown in several studies and has the potential to encode synaptic signals at the site of origin and decode them in the nucleus. In this review, we summarize the knowledge about the properties of the synapto-nuclear messenger protein Jacob with special emphasis on a putative role in hippocampal neuronal plasticity. We will elaborate on the interactome of Jacob, the signals that control synapto-nuclear trafficking, the mechanisms of transport, and the potential nuclear function. In addition, we will address the organization of the Jacob/*NSMF* gene, its origin and we will summarize the evidence for the existence of splice isoforms and their expression pattern.

## Introduction

The complex morphology of neuronal cells poses a major challenge to integrate and link synaptic signals arising on distant dendritic branches to nuclear gene expression. This is a rather complex process and may require different modes of regulation. Several excitation-transcription coupling pathways are triggered downstream of N-methyl-D-aspartate receptors (NMDARs) and L-type voltage-gated Ca^2+^ channels. Activation of Ca^2+^ signaling in the neuronal soma includes modulation of calcium release from intracellular calcium stores, backpropagating action potentials, somatic propagation of dendritic Ca^2+^ spikes that are independent of action potentials (Cohen and Greenberg, 2008; Hardingham and Bading, 2010; West and Greenberg, 2011; Bading, 2013; Morris, 2013; Volianskis et al., 2015; Wild et al., 2019). Thus, not only nuclear Ca^2+^ -waves elicited by NMDAR and L-type voltage-gated Ca^2+^ channels are instrumental in the control of gene expression but also soma-to-nucleus signaling is regulated by Ca^2+^ and might, for instance, control the nuclear import of transcription factors (Wild et al., 2019). However, it is questionable that calcium signals alone can elicit a specific nuclear response that precisely encodes signals coming from distinct receptors located on different dendritic sites and activated by diverse stimuli.

Long-distance transport of macromolecular protein signaling complexes can potentially provide a more precise means of encoding and transducing different types of synaptic activation to the nucleus. The type of information might include for instance the localization and a number of activated NMDAR and published evidence suggests that synapto-nuclear protein messenger might convey this type of information to the nucleus where it is translated in distinct long-lasting changes in gene expression (Dieterich et al., 2008; Lai et al., 2008; Fainzilber et al., 2011; Ch’ng et al., 2012; Karpova et al., 2013; Zhai et al., 2013; Dinamarca et al., 2016; Kravchick et al., 2016).

The prevalent model of activity-induced protein transport from synapses to the nucleus implies the binding of a nuclear localization signal (NLS) in synapto-nuclear protein messengers with one of the importin-α family members in response to synaptic activation. This is followed by binding to importin-β and association with a dynein motor that eventually mediates the transport of the protein complex along microtubule toward the nucleus (Cingolani et al., 1999; Fainzilber et al., 2011; Ch’ng and Martin, 2011; Kaushik et al., 2014; Lever et al., 2015; Panayotis et al., 2015; Lee et al., 2020).

## Jacob Signalosome and Its Function in the Regulation of Gene Expression

**FIGURE 1:**
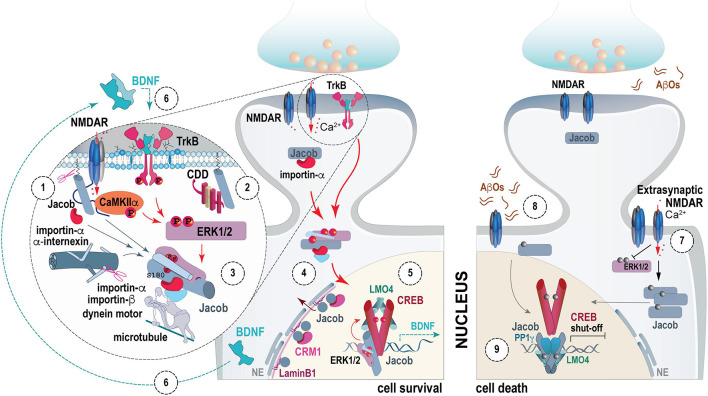
Molecular mechanisms underlying synapse-to-nucleus trafficking of Jacob and its nuclear function. N-terminal myristoylation is a prerequisite for the extranuclear localization of Jacob. In synapses, Jacob associates with the GluN2B-containing NMDA receptor complex as well as CaMKII-α (1). The synaptic localization is regulated by the neuronal Ca^2+^ sensor protein Caldendrin (2) that competes with importins for Jacob binding. NMDAR activation leads to calpain-mediated cleavage of the myristoylated part of the protein and releases Jacob from the plasma membrane. Concomitantly, synaptic GluN2B-containing NMDAR activity leads to CaMKII-α dependent activation of ERK1/2, subsequent phosphorylation of Jacob at S180 and formation of a stable trimeric complex between phosphorylated Jacob, active ERK1/2 and the proteolytically cleaved fragment of the neuronal filament α-internexin (3) which protects pJacob and pERK1/2 against phosphatase activity during retrograde transport to the nucleus but likely also in the nucleus. Long-distance transport of the Jacob signalosome involves its association with importin-α, importin-β, and, subsequently the molecular motor dynein that moves along microtubules in a retrograde direction. GluN2B-containing NMDA receptor activity mediates the association of Jacob with the inner nuclear membrane where it transiently binds to LaminB1 (4). The association with the canonical CRM1-RanGTP-dependent export complex defines its nuclear residing time. In the nucleus, the Jacob signalosome associates with the CREB complex and results in its sustained activation by docking the active ERK1/2 in its close vicinity (5). This, in turn, promotes CREB-dependent gene expression of plasticity-related genes like *Bdnf*. In early development, BDNF induces the nuclear accumulation of phosphorylated Jacob in an NMDAR-dependent manner, which results in increased phosphorylation of CREB and enhanced CREB-dependent *Bdnf* gene expression in a positive feedback loop (6). Activation of extrasynaptic NMDARs by NMDA (7) or AβOs (8) does not lead to phosphorylation of ERK1/2 or Jacob. Nevertheless, the non-phosphorylated protein translocates to the nucleus (9) piggyback with the CREB phosphatase, PP1. In addition, it displaces CREB from the transcriptional co-activator LMO4 leading to CREB shut-off. CDD, Caldendrin; NE, nuclear envelope; red circle, phosphorylation; gray circle, unphosphorylated site; TrkB, Tropomyosin receptor kinase B; NMDAR, N-methyl-D-aspartate receptor; LMO4, LIM domain only 4; CREB, cAMP response element-binding protein; BDNF, brain-derived neurotrophic factor; CRM1, chromosomal maintenance 1; ERK1/2, Extracellular signal-regulated protein kinases 1 and 2; PP1, protein phosphatase 1. Scissors indicate cleavage.

Jacob, the protein encoded by the *NSMF* gene, is a synapto-nuclear messenger that encodes and transduces NMDAR signals to the nucleus (Karpova et al., 2013). Jacob assembles a signalosome likely in close vicinity to NMDAR and following long-distance transport docks this signalosome to the transcription factor cAMP-responsive element-binding protein (CREB, Figure 1; Dieterich et al., 2008; Karpova et al., 2013; Spilker et al., 2016b; Grochowska et al., 2017).

NMDARs have been implicated in synaptic plasticity, learning and memory, cell survival signaling, but also in neurodegeneration and excitotoxicity (Paoletti et al., 2013; Pagano et al., 2021). A prevailing hypothesis in the field suggests that the opposing functions of NMDARs attribute to their subcellular localization and subunit composition. Signaling downstream of synaptic and extrasynaptic NMDARs is tightly coupled with the transcriptional activity of CREB where activation of synaptic NMDARs promotes its sustained phosphorylation at a crucial serine residue at position 133 and subsequent expression of plasticity-related genes (Hardingham and Bading, 2010). Therefore, synaptic NMDARs are crucial for plasticity processes like the expression of long-term potentiation (LTP), memory encoding and consolidation (Shimizu et al., 2000; Thompson et al., 2004; Jordan et al., 2007; Dieterich et al., 2008; Jeffrey et al., 2009; Jordan and Kreutz, 2009; Hardingham and Bading, 2010; Kaufman et al., 2012; Morris, 2013; Papouin and Oliet, 2014; Dinamarca et al., 2016; Bading, 2017). Conversely, activation of extrasynaptic GluN2B-containing NMDARs leads to sustained dephosphorylation of CREB, also known as CREB shut-off, rendering CREB transcriptionally inactive (Hardingham et al., 2002; Hardingham and Bading, 2010; Rönicke et al., 2011; Bading, 2013).

Loss of CREB-dependent pro-survival gene expression after extrasynaptic NMDAR activation seems to precede cell death and neurodegeneration in diseases like Alzheimer’s disease (AD) and Huntington’s disease (HD) (Shankar et al., 2007; Milnerwood et al., 2010; Malinow, 2012; Kessels et al., 2013; Papouin and Oliet, 2014; Parsons and Raymond, 2014; Wild et al., 2015; Carvajal et al., 2016; Bading, 2017; Grochowska et al., 2017; Marcello et al., 2018; Parra-Damas and Saura, 2019). Especially in AD, dysregulation of CREB-dependent gene expression apparently plays a role in the onset of the disease and early synaptic dysfunction (Saura and Cardinaux, 2017).

The synaptic localization of Jacob is mediated in part by its association with the plasma membrane via a myristoyl group attached to its N-terminal glycine residue (Dieterich et al., 2008; Karpova et al., 2013). In addition, Jacob associates with calmodulin-dependent protein kinase II-α (CamKII-α) and with the C-terminal tail of the GluN2B subunit of NMDAR (Figure 1.1; Dieterich et al., 2008; Dinamarca et al., 2016; Melgarejo da Rosa et al., 2016). In spine synapses, the neuronal Ca^2+^ sensor protein Caldendrin binds to a central IQ-like motif of Jacob and thereby masks a bipartite NLS involved in importin-α binding (Figure 1.2; Dieterich et al., 2008). Interestingly, Caldendrin is like Jacob particularly prominent in larger mushroom-like dendritic spines that are tightly sealed by the spine neck and show highly compartmentalized Ca^2+^-responses (Seidenbecher et al., 1998; Dieterich et al., 2008; Mikhaylova et al., 2018). Binding of Jacob to Caldendrin presumably keeps the protein in spines until all steps of signalosome formation are accomplished. Hence, the influx of Ca^2+^ through NMDARs is crucial for the release of Jacob from synaptic sites since it activates the protease calpain, which in turn cleaves the myristoylated N-terminal part releasing the protein from the plasma membrane (Figure 1.1; Kindler et al., 2009; Karpova et al., 2013).

Ca^2+^-influx through GluN2B-containing synaptic NMDARs leads to the subsequent activation of Extracellular Signal-Regulated protein Kinases 1 and 2 (ERK1/2) via Ca^2+^/CaMKII-α (El Gaamouch et al., 2012). This results in phosphorylation of Jacob at a crucial serine residue at position 180 (S180/Figure 1.3; Karpova et al., 2013; Melgarejo da Rosa et al., 2016). Concomitantly, Jacob phosphorylated at S180 and active ERK1/2 assemble a trimeric complex with calpain cleaved fragments of the intermediate neuronal filament α-internexin. The binding of α-internexin further stabilizes the Jacob/ERK1/2 complex and protects it from the cytosolic phosphatase-rich environment of neurons *en route* to the nucleus (Karpova et al., 2013).

The adaptor protein importin-α1/Rich1 (encoded by *KPNA2* gene), which links cargo to importin-β1, directly interacts with the NLS of Jacob and this interaction is essential for transport (Figure 1.3; Dieterich et al., 2008; Karpova et al., 2013). Likely, that this complex is already formed at synapses since both, importin-α1 and importin-β1 are present at spines and distal dendrites and have been shown to translocate to the nucleus in response to synaptic activity (Thompson et al., 2004). There are at least seven importin-α family members expressed in the mammalian brain (Kelley et al., 2010), but whether they compete for binding to Jacob’s NLS is currently unclear. Potentially, multiple combinations of importin-α/importin-β complexes exist and might represent the importin code of synapto-nuclear protein messengers that depicts the grand cargo specificity (Lever et al., 2015). Finally, the Jacob signalosome associates with the molecular motor cytoplasmic dynein that is instrumental for trafficking of the protein complex along microtubules toward the nucleus (Karpova et al., 2013).

Following NMDAR-dependent nuclear import, Jacob transiently associates with the inner nuclear membrane (Figure 1.4) by direct interaction with the nuclear lamina protein LaminB1 and the nuclear export adaptor chromosomal maintenance 1 (CRM1; Samer et al., 2021). At present, it is unclear whether the nuclear lamina merely provides a docking site for an intermediate step relevant for either subsequent redistribution of Jacob to nuclear target sites or its nuclear export (Samer et al., 2021).

A prominent nuclear target of Jacob is the transcription factor CREB (Figure 1.5). Of note, the direct interaction of Jacob with CREB does not depend on the phosphorylation of S180. In response to synaptic NMDAR activation, Jacob phosphorylated at S180 accumulates in the nucleus (Dieterich et al., 2008; Karpova et al., 2013; Spilker et al., 2016b; Grochowska et al., 2017) where it then binds CREB (Karpova et al., 2013; Grochowska et al., 2020).

Association with Jacob promotes CREB phosphorylation at S133 in an ERK-dependent manner and thereby links synaptic NMDAR activity to CREB-dependent gene expression related to synaptic plasticity (Figure 1.6; Karpova et al., 2013; Spilker et al., 2016b).

Interestingly, Jacob is imported to the nucleus in rat hippocampal slices and neuronal cultures only after induction of NMDAR-dependent LTP but not Ltd. (Behnisch et al., 2011; Yuanxiang et al., 2014; Melgarejo da Rosa et al., 2016). Of note, it accumulates in the nucleus already within 30 min after LTP induction, a time window critical of activity-induced gene expression required for long-lasting LTP expression (Frey et al., 1996; Behnisch et al., 2011). Accordingly, Jacob/*NSMF* knockout mice display impaired expression of Schaffer collateral LTP (Spilker et al., 2016b), emphasizing a potential role of the messenger protein in transmitting LTP-related signals from synapse to nucleus.

Synaptic NMDAR signals are key for Jacob nuclear import in mature neurons. However, BDNF, whose expression is regulated via the NMDAR-Jacob-CREB pathway, can also promote the accumulation of S180 phosphorylated Jacob in the nucleus in neuronal development as part of a positive feedback loop that drives BDNF expression in a CREB-dependent manner (Figure 1.6; Spilker et al., 2016a,b). BDNF-dependent translocation of Jacob to the nucleus appears to play a critical role in hippocampal development. Accordingly, Jacob/*NSMF* knockout mice display hippocampal dysplasia that is characterized by reduced complexity of the dendritic tree of pyramidal neurons, a reduced number of synaptic contacts, an altered catechol- and monoaminergic innervation, as well as reduced BDNF expression and impaired nuclear ERK1/2 and CREB signaling (Spilker et al., 2016a,b). Structural alterations in the hippocampus correlate with functional deficits related to learning and memory. Particularly, Jacob/*NSMF* knockout mice show impaired contextual fear conditioning and object recognition memory, behavioral tasks that are sensitive to hippocampal dysfunction (Spilker et al., 2016a,b).

Activation of extrasynaptic NMDARs leads to the formation and translocation of a different Jacob transport complex (Dieterich et al., 2008; Karpova et al., 2013; Grochowska et al., 2020). Several lines of evidence indicate that extrasynaptic NMDAR activity evoked by the block of synaptic NMDARs and subsequent treatment with NMDA induces dephosphorylation of ERK1/2 and Jacob (Figure 1.7; Ivanov et al., 2006;Rönicke et al., 2011; Karpova et al., 2013; Gomes et al., 2014; Grochowska et al., 2017; Grochowska et al., 2020) and triggers CREB shut-off resulting in synaptic dysfunction, synapse loss and subsequent cell death (Hardingham and Bading, 2010; Yan et al., 2020). Soluble amyloid-β oligomers (AβOs) are causative agents underlying the onset and progression of AD (Selkoe and Hardy, 2016; Cline et al., 2018). It was shown that different AβOs species, Aβ1-42 and Aβ25-35, act on extrasynaptic NMDARs and drive non-phosphorylated Jacob in the nucleus which triggers CREB shut-off (Figure 1.8; Rönicke et al., 2011; Gomes et al., 2014; Grochowska et al., 2017, 2020). Jacob seems to play a role in extrasynaptic NMDAR signaling linked to neurodegenerative disorders and interrupted CREB-dependent gene expression at the early stage of AD pathology (Rönicke et al., 2011; Gomes et al., 2014; Grochowska et al., 2017, 2020). Along these lines, Jacob protein knockdown abolished AβOs-induced CREB shut-off and, concurrently, ameliorated neuronal loss in the CA1 area of the hippocampus in a double transgenic AD mouse line lacking the *NSMF* gene (Grochowska et al., 2020).

In mature neurons, non-phosphorylated nuclear Jacob preferentially binds to LIM domain Only 4 (LMO4), a CREB coactivator, replaces LMO4 from the transcription factor complex and impairs its transcriptional activity (Figure 1.9; Grochowska et al., 2020). Furthermore, nuclear non-phosphorylated Jacob docks the protein phosphatase 1 (PP1) to CREB sites further promoting its transcriptional inactivation (Hardingham and Bading, 2010; Grochowska et al., 2017, 2020). Interestingly, both CREB shut-off and nuclear import of non-phosphorylated Jacob cannot be induced in young neuronal cultures, less than 9 days *in vitro* (DIV), which indicates that this type of signaling requires a certain level of network maturation and substantial expression of GluN2B-containing NMDAR at extrasynaptic sites (Sheng et al., 1994; Sala et al., 2000; Hardingham et al., 2002; Behnisch et al., 2011).

Although extensively studied in the context of neurodegenerative diseases, the extrasynaptic NMDAR pathway has also been shown to play an important role in classical synaptic memory mechanisms (Henneberger et al., 2020; Herde et al., 2020) and behavior (Homiack et al., 2017). Using a synthetic predator odor 2,5-dihydro-2,4,5-trimethylthiazoline (TMT) exposure protocol as a model of post-traumatic stress disorder (PTSD) in rats, Homiack et al. (2017) have shown that TMT exposure reduced phosphorylation of CREB in male, but not female rats. Moreover, reduced ERK1/2 phosphorylation together with an increase in nuclear accumulation of Jacob was also found in the hippocampus of male rats, strong evidence of the activation of the CREB shut-off pathway. The differential signaling cascade activation between sexes warrants further investigation, given that TMT exposure produces the same outcome in both sexes at the behavioral level.

## Cell-Type Specific Expression Patterns, *NSMF* Gene Structure and Jacob Splice Isoforms

Studies on the function of Jacob as synapto-nuclear protein messenger have been mainly done in pyramidal neurons of the hippocampus and cortex (Dieterich et al., 2008; Kindler et al., 2009; Behnisch et al., 2011; Karpova et al., 2013; Mikhaylova et al., 2014). However, Jacob is expressed in neurons of various brain regions (Mikhaylova et al., 2014) and it would be interesting to learn whether it has a similar function in other neuronal cell types. In pyramidal neurons the protein is present in distal dendrites and axons where it localizes to pre- and postsynaptic sites with a clear enrichment at the postsynaptic density (PSD) as confirmed by fluorescence and electron microscopy (EM) as well as by subcellular fractionation experiments (Dieterich et al., 2008; Mikhaylova et al., 2014). Jacob is prominently present in nuclei of pyramidal neurons where it associates with distinct nuclear loci including the inner nuclear membrane (Samer et al., 2021). Although Jacob is expressed in Parvalbumin-, Calbindin-, and Calretinin-positive interneurons of the hippocampus as well as in medium spiny neurons (MSN) of the striatum, principal differences in the expression pattern of Jacob between excitatory and inhibitory neurons concern the synaptic localization of the protein, although inhibitory neurons show a somato-dendritic distribution of Jacob. Stimulated emission depletion (STED) imaging revealed that the protein is absent at inhibitory shaft synapses and expressed at very low levels only in a subset of cortico-striatal synapses of medium spiny neurons (Mikhaylova et al., 2014; Bär, 2015). Interestingly, however, the nuclear localization of Jacob is very similar between inhibitory and excitatory neurons (Dieterich et al., 2008; Behnisch et al., 2011; Mikhaylova et al., 2014).

Jacob/*NSMF* expression appears to be developmentally regulated with the highest mRNA levels during synaptogenesis, between the second and the third postnatal week (Dieterich et al., 2008; Kindler et al., 2009; Bär, 2015). This period also correlates with an increase in Jacob protein expression. The Jacob/*NSMF* mRNA shows a prominent dendritic localization in the hippocampus (Kindler et al., 2009). Like other proteins that might be locally translated in dendrites, the Jacob/*NSMF* mRNA harbors a dendritic targeting element (DTE) that is part of the 3‘UTR region of the transcript (Kindler et al., 2009). Further evidence for local translation of Jacob’s dendritic mRNA in cortical neurons comes from a study employing SynapTRAP, a synaptoneurosomal fractionation followed by translating ribosome affinity purification (Ouwenga et al., 2017). Like all dendritic mRNAs isolated in this study, the Jacob mRNA had a disproportionately longer length and was enriched for Fragile-X mental retardation protein (FMRP) binding. Interestingly, Jacob is an FMRP target (Kindler et al., 2009) and multiplexed error-robust fluorescence *in situ* hybridization (MERFISH) revealed that in comparison to the cellular distribution of ∼4200 RNA species in hippocampal primary cultures Jacob/*NSMF* mRNA expression in distal dendrites belongs to the top 10% of transcripts showing the highest dendrite-to-soma transcript ratio (Wang et al., 2020). Local translation likely replenishes the synaptic protein pool following synapse-to-nucleus transport. Unfortunately, it is at present unclear whether only certain transcripts containing the NLS and the synaptic targeting element are preferentially translated in dendrites (see also below).

**FIGURE 2:**
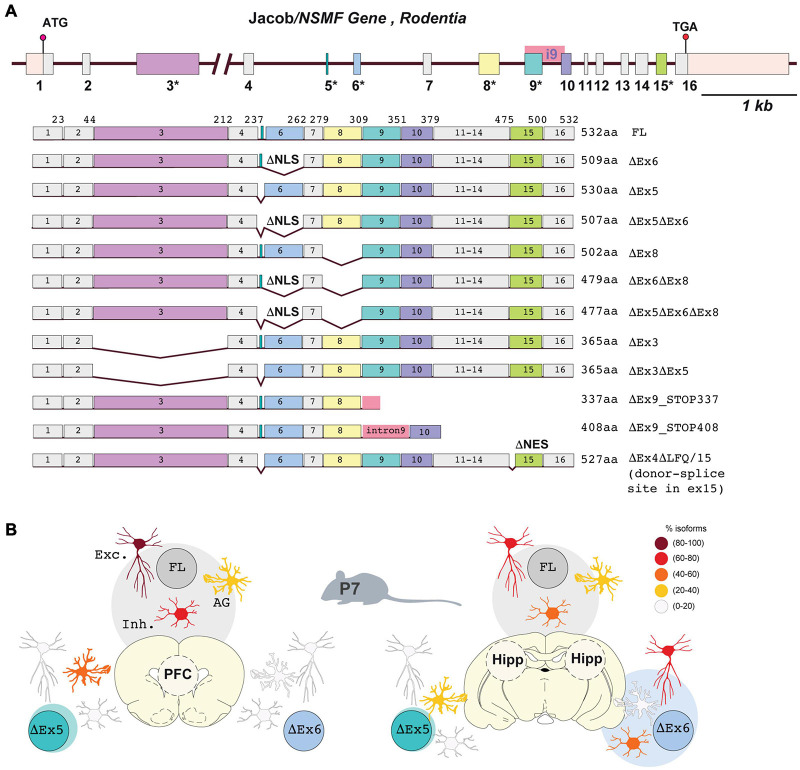
Jacob*/NSMF* gene in rodents comprises 16 exons from which at least 5 are alternatively spliced. **(A)** Schematic representation of Jacob/*NSMF* gene structure (modified from Spilker et al., 2016a) and experimentally confirmed alternatively spliced mRNA variants resulting in various protein isoforms. Numbers with an asterisk represents alternatively spliced exons. Exons that are not spliced are indicated in gray. Isoform name and its length in amino acids (aa) are indicated in the right panel. **(B)** Schematic representation of the cell type-specific relative expression pattern of Jacob/*NSMF* splice variants based on single-cell isoform RNA sequencing and genePlot analysis. Indicated are excitatory neurons (Exc.), inhibitory neurons (Inh.) and astrocytic glia (AG). Color code from white-to-dark red indicates relative amounts of mRNA transcripts in the prefrontal cortex (PFC) and hippocampus (Hipp) in early development (P7 mouse brain). Shaded circles indicate the relative abundance of a particular isoform vs. others within the brain region.

The Jacob*/NSMF* gene structure is rather complex with 16 exons (Figure 2A) and high sequence conservation between mammalian species. It has been shown that at least 5 out of 16 exons of the Jacob/*NSMF gene* can be alternatively spliced (Figure 2A; Miura et al., 2004; Dieterich et al., 2008; Kindler et al., 2009; Miura et al., 2013; Quaynor et al., 2014;Joglekar et al., 2021). The existence of many Jacob/*NSMF* isoforms are predicted, but not all of them are experimentally confirmed and not much information is available about brain region- and cell type-specific expression of splice variants and regulation of expression in development. The most recent version of the database from the National Center for Biotechnology Information (NCBI, 09.2021) indicates 13 isoforms for mouse Jacob/*NSMF* (*M. musculus, Gene ID: 56876*) out of which 8 are validated. For the human Jacob/*NSMF* gene (*H. sapiens, Gene ID: 26012*) 5 out of 10 transcripts predicted by automated computational analysis are confirmed.

Experimental studies on Jacob/*NSMF* mRNA expression have shown that several Jacob/*NSMF* transcripts are present in the mouse, rat, and human brain (Dieterich et al., 2008; Kindler et al., 2009; Xu et al., 2011). Single-cell isoform RNA sequencing in the prefrontal cortex (PFC) and hippocampus of the mouse brain at postnatal day 7 suggests that Jacob/*NSMF* is widely expressed in inhibitory and excitatory neurons in both brain regions (Figure 2B; Joglekar et al., 2021). Interestingly, the expression of an isoform lacking exon 6 appeared to be restricted to hippocampal neurons. This 69 nucleotide (nt)-long exon encodes a nuclear localization signal and part of the IQ- domain that forms the interface for Caldendrin binding. Therefore, alternative splicing of this specific exon may affect the synapto-nuclear distribution of Jacob and its nuclear function. Altogether, the expression of the full length (FL) isoform in the PFC is relatively higher in comparison to the hippocampus due to the absence of other isoforms. This poses the PFC as a well-suited region for the study of the synapto-nuclear function of the protein. A Jacob transcript lacking the 6 nt-long exon 5 was detected only in glial cells of the PFC in P7 mouse brains (Figure 2B; Joglekar et al., 2021), which is at variance with the lack of Jacob protein expression in astro- and microglia in adult rat brain (Mikhaylova et al., 2014).

## Evolutionary Conservation of the Coding Sequence of the Jacob*/NSMF* Gene

Most studies on the synapto-nuclear messenger function of Jacob were performed with mammalian species (Dieterich et al., 2008; Kindler et al., 2009; Behnisch et al., 2011; Karpova et al., 2013; Melgarejo da Rosa et al., 2016; Spilker et al., 2016b; Grochowska et al., 2017). However, the *NSMF* gene has also been found in zebrafish [called *nasal embryonic LHRH factor (NELF*); Kramer and Wray, 2000; Palevitch et al., 2009]. A database search shows that the *NSMF* gene is present in vertebrates comprising all groups of the taxon euteleostomi, namely Tetrapoda and Osteichthyes (bony fish). The recently updated NCBI database (09.2021) contains entries for *NSMF* genes in 280 species. Examples include mammals (e.g., *R. norvegicus, M. musculus, H. sapiens, P. troglodytes)* but also birds (e.g., *G. gallus*), reptiles (e.g., *Chr. picta*), amphibians (e.g., *X. tropicalis*), and fish (e.g., *D. rerio*) (Table 1). An extended Ensembl (release 104) genome database project (Howe et al., 2020) search identified the *NSMF* gene sequence in a scaffold of the sea lamprey *(P. marinus).* Although the total gene size varies from 8.5 kb in mouse to approximately 80 kb in zebrafish the exon organization is highly conserved with an exception for the lowest vertebrate *P. marinus* where exon boundaries are shifted, and the number of Jacob coding regions is 17. It is striking that the homology in the Jacob amino acid sequence between *R. norvegicus, G. gallus, D. rerio* (*NSMF a* gene) and *P. marinus* is very high in its C-terminus, especially within the regions encoded by exons 10-16 where most species show sequence identity. The differences in amino acid sequence between different species concern largely parts of the protein encoded by exon 3 (which is alternatively spliced), as well as exons 8 and 9. Altogether, these findings suggest that Jacob is expressed throughout all vertebrates (Table 1) with the highest conservation within its C-terminus. Vice versa, no invertebrate ortholog of Jacob was found using diverse tools [i.e., NCBI Expressed Sequence Tags (EST) search^1^]. Basic Local Alignment Search Tool (BLAST) of different exons of diverse species, Ensembl genome database project (release 104; Howe et al., 2020), NCBI HomoloGene).

**TABLE 1:** Accession numbers of Jacob protein and Jacob/*NSMF* gene sequences reviewed for conservation.

**Species**	**Common name**	**Genomic, cDNA or mRNA sequence**	**Protein sequence**
*H. sapiens*	Human	NM_001130969.3 GeneID 26012 ENST00000371475	NP_001124441.1
*R. norvegicus*	Common rat	NM_057190.2 GeneID ENSRNOT00000061303	NP_476538.2 Uniprot Q9EPI6
*M. musculus*	House mouse	NM_001039386.1 GeneID 56876 ENSMUST00000100334	NP_001034475.1 Uniprot Q99NF2
*G. gallus*	Chicken	GeneID 417260 ENSGALG00000008681 XM_015279659.3	XP_015135145.1
*D. rerio*	Zebrafish	factor a gene ID 555195 ZDB-GENE-091204-32 7955.ENSDARP00000117074 ENSDARG00000060025 XM_009295251.3	Directly translated from gene sequence XP_009293526.1
*D. rerio*	Zebrafish	Factor b Gene ID: 569891 XM_021476298.1 NP_001143901.1 ZDB-GENE-080603-4 7955.ENSDARP00000108693 ENSDARG00000101234	XP_021331973.1 NP_001137373.1
*P. marinus*	Sea lamprey	ENSPMAT00000004778.1 GL476904	Directly translated from cDNA

## Jacob Interactome and Conservation of Binding Motifs

**FIGURE 3:**
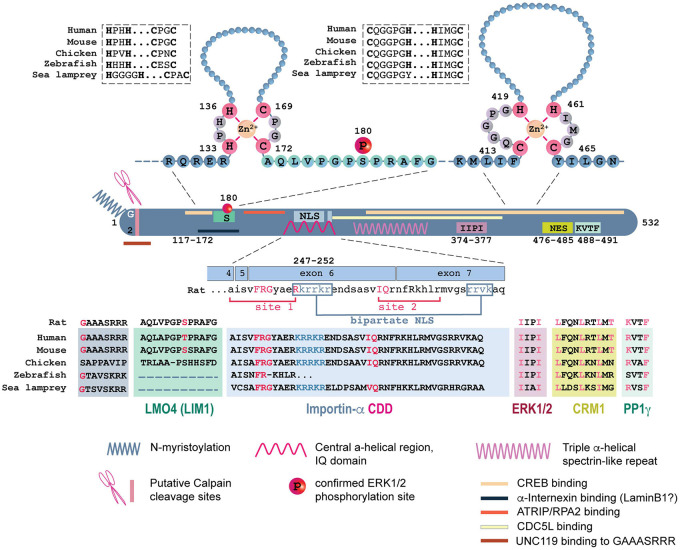
Jacob interactome and motif conservation. Schematic representation of the binding motifs and binding regions for multiple confirmed interactors (represented by shaded boxes). The upper panel represents two well-conserved putative zinc finger domains (HH-CC and CH-HC consensus, *modified from* Xu et al., 2010). Cysteine or histidine residues directly binding to zinc ions are indicated in pink. Sequence conservation for the zinc finger domains is indicated in bold inside the boxes. Zebrafish Jacob (factor a) sequence is used for the panel. Amino acids encompassing the LMO4 (LIM1) binding site located directly after the first zinc finger domain are indicated in green. Color bars indicate interacting partners. Color boxes indicate motifs. Crucial amino acids residues within the motives are indicated in red. RPA2, replication protein A 2; ATR, ataxia telangiectasia and Rad3-related protein; CDC5L, Cell Division Cycle 5 Like; ATRIP, ATR interacting protein; CDD, Caldendrin; UNC119, solubilizing factor.

In recent years several motifs and binding partners of Jacob have been identified (Figure 3; Dieterich et al., 2008; Karpova et al., 2013; Melgarejo da Rosa et al., 2016; Grochowska et al., 2020; Ju et al., 2021). The protein is largely unstructured, which is a common feature of adaptor proteins that assemble a signalosome. The following analysis of experimentally confirmed main motifs in the primary sequence of Jacob and its conservation between species is based on public database searches (Archive Ensembl release 104; Howe et al., 2020).

Rodent Jacob is a 532 amino acid (aa) protein (Figure 3) with no known domain organization, a predicted disordered secondary structure, and many potential phosphorylation sites (37 out of 51 serines; Bär, 2015; Spilker et al., 2016a). Phosphorylation of Jacob by ERK1/2 at S180 (T178 in the human sequence) is experimentally confirmed and it is well conserved among mammals (Figure 3; Karpova et al., 2013). On the other hand, the ERK1/2 binding motif (IxxI) encoded by exon 10 is highly conserved throughout all species, potentially allowing Jacob phosphorylation and raising the possibility of Jacob signalosome formation in other species than mammals (Figure 3). A functional N-terminal myristoylation motif is only present in mammals, although the glycine residue at the N-terminus is also present in zebrafish and sea lamprey. The functional relevance of this modification in mediating membrane attachment was confirmed by site-directed mutagenesis where overexpression of Jacob lacking the crucial glycine at position 2 resulted in its exclusive nuclear localization (Kramer and Wray, 2000; Dieterich et al., 2008; Karpova et al., 2013). Recently, a high-affinity interaction of an N-terminal Jacob peptide (GAAASRRR) with solubilizing factor UNC119 has been described (Figure 3; Yelland et al., 2021).

The binding of Jacob to Caldendrin relies on its central α-helix (Dieterich et al., 2008) that is encoded by a region spanning the end of exon 4 until the middle of exon 7 (Figure 3, site 1 and site 2). Particularly, a phenylalanine at position 241, that provides anchoring of the central α-helical region into the hydrophobic pocket of Caldendrin is essential for the interaction (Landwehr et al., 2003; Dieterich et al., 2008). The sequences available in the NCBI database indicate high sequence homology and the conservation of the F241 throughout all species, whereas the entire motif shows substantial variability in zebrafish (Figure 3). Additionally, exon 5 that in mammals codes for the amino acids isoleucine (I) and serine (S) that are known to enhance binding of Jacob to Caldendrin (Dieterich, 2003), is present throughout euteleostomi.

Exon 6 and exon 7 of the *NSMF* gene encode the bipartite NLS (Figure 3). All investigated species except zebrafish harbor an NLS in their primary structure. The nuclear-cytoplasmic shuttling of Jacob is also controlled by a nuclear export signal (NES) that is encoded by exon 15 and that serves as the binding interface for CRM1 (Samer et al., 2021). This consensus motif is identical in all mammals (Figure 3), however, CRM1 binding might be altered due to variations in the last amino acid within the motif in birds, zebrafish, and sea lamprey.

Two putative zinc finger domains with HH-CC motif (aa 133–172, encoded by the exon 3) and CH-HC motif (aa 413–465, spanning the exon 12–14) in which two cysteine and two histidine residues coordinate zinc binding were predicted in Jacob sequence (Xu et al., 2011). These common motifs largely define protein-DNA interaction, but can also mediate protein-RNA interaction and protein-protein interaction including dimerization (Mackay and Crossley, 1998; McCarty et al., 2003; Cassandri et al., 2017). Additional sequence alignments revealed high sequence conservation in these regions among human, rat, mouse, chicken, and zebrafish with the exception of sea lamprey (Figure 3). CREB directly binds to Jacob at its N-terminus (117–172 aa identified as the minimal region) and at its C-terminus containing putative zinc finger domains. The transcriptional co-activator of CREB, LMO4, associates with Jacob immediately after (172–228 aa) the first HH-CC zinc finger domain in a manner depending upon S180 phosphorylation (Grochowska et al., 2020). This region is only conserved in mammals (Figure 3) and it is therefore, likely that the Janus-face of the protein in terms of CREB-phosphorylation has only emerged relatively late during evolution.

## Concluding Remarks and Future Perspectives

Several studies support the idea that Jacob serves as a mobile signaling hub that by docking to nuclear targets might induce long-lasting changes in gene expression.

At present, not much is known about how long-distance transport of other synapto-nuclear protein messengers might converge to CREB signaling. The most prominent candidate for such a role is probably the nuclear translocation of CREB-Regulated Transcriptional Co-activator (CRTC1). Interestingly, both CRTC1 and Jacob bind to the bZip domain of CREB (Conkright et al., 2003; Ch’ng et al., 2012; Grochowska et al., 2020). This opens the possibility that both proteins either will compete for CREB binding or might functionally interact in one dimeric CREB complex. Alternatively, transport of both proteins from synapse-to-nucleus might encode different information that might affect the expression of different target genes. Along these lines, it is also conceivable that Jacob or CRTC1 are only recruited to the transcription machinery at different gene promoters to initiate CREB-dependent gene transcription and, therefore, will not compete for binding to CREB at all.

The exon/intron structure and the amino acid sequence are highly conserved in mammalian species, although gene size varies due to different sizes of introns. The high conservation of the C-terminus compared to the N-terminus is striking. Since the majority of known Jacob functions are linked to its N-terminus, the role of splice isoforms lacking this protein part is hard to predict. Furthermore, we undertook the effort of describing the plethora of isoforms to stress that we only begin to understand the role of the gene and that there may be additional functions.

Interestingly, the N-terminus is the region that has the lowest evolutionary conservation and disordered structure without clear domains. It is possible, that only phosphorylation of some of the numerous predicted sites and/or binding to interacting partners stabilizes the protein, which could be linked to specific signaling events. One might also speculate that many aspects of NMDAR signaling to the nucleus might have evolved relatively late, possibly with the evolution of spine synapses and regulated dynamics of NMDAR localization at synaptic and extrasynaptic sites.

Another important aspect of synapto-nuclear communication is the retrograde transport from presynaptic sites along the axon. This has been well-documented for importins upon axonal injury (Hanz et al., 2003; Perlson et al., 2005), and also the nuclear import of the presynaptic signaling molecule CtBP1 has been described (Ivanova et al., 2015). We could show the expression of Jacob at excitatory synapses not only on post-, but also presynaptic sites (Mikhaylova et al., 2014), although further confirmation with super-resolution imaging is favorable. Nonetheless, we found Jacob expression along axons in mossy fibers, making a presynaptic localization very likely. A possible function of Jacob in presynapse-to-nucleus communication is therefore, conceivable.

## Author Contributions

KMG, JB, GMG, MRK, and AK wrote the manuscript. AK prepared the figures. All authors commented on the manuscript.

## Conflict of Interest

The authors declare that the research was conducted in the absence of any commercial or financial relationships that could be construed as a potential conflict of interest.

## Publisher’s Note

All claims expressed in this article are solely those of the authors and do not necessarily represent those of their affiliated organizations, or those of the publisher, the editors and the reviewers. Any product that may be evaluated in this article, or claim that may be made by its manufacturer, is not guaranteed or endorsed by the publisher.

## Footnotes

1
https://www.ncbi.nlm.nih.gov/nuccore/
